# Unilateral Agenesis of the Internal Carotid Artery: A Report of Two Rare Cases: Unilateral ICA Agenesis

**DOI:** 10.31661/gmj.v11i.2318

**Published:** 2022-12-25

**Authors:** Seyed Kamaledin Hadei, Mahdi Ramezani, Reza Taherian

**Affiliations:** ^1^ Department of Radiology, School of Medicine, Hamadan University of Medical Sciences, Hamadan, Iran; ^2^ Department of Anatomy, School of Medicine, Hamadan University of Medical Sciences, Hamadan, Iran

**Keywords:** Cerebrovascular Insufficiency, Circle of Willis, Agenesis, Internal Carotid Artery

## Abstract

**Background:**

Internal carotid artery (ICA) agenesis occurs when one or both of the blood vessels that supply blood to the brain do not develop. Congenital agenesis of the ICA rarely occurs. It is usually asymptomatic but may sometimes associate with neurological symptoms such as migraine and pulsatile tinnitus. Moreover, differentiating it from occlusion of ICA is important in patients with stroke.

**Case Report:**

We report two cases (63-years-old man and 69-year-old woman) of asymptomatic unilateral ICA agenesis who were referred to our cardiovascular hospital for coronary artery bypass graft. Due to a suspicious history of transient ischemic attack, the patients underwent carotid ultrasonography. With findings suggestive of unilateral ICA agenesis at color Doppler, patients underwent computed tomography angiography that confirmed the diagnosis.

**Conclusion:**

Suspecting ICA agenesis at color Doppler imaging of the neck and differentiating it from occluded ICA at CT angiography is important for correct diagnosis and management of the patients.

## Introduction

Unilateral agenesis of the internal carotid artery (ICA) is a rare entity. ICA agenesis occurs when one or both ICA do not develop [1]. However, ICA agenesis could not present with symptoms because collateral pathways carry the blood to the brain [1][2]. However, some individuals may have symptoms such as headaches, blurred vision, paralysis of some cranial nerves, recurrent seizures, and hemiparesis [2][3]. Also, some individuals with ICA agenesis may have other malformations involving the blood vessels, face, and/or ears. This typically occurs on the same side of the body as the ICA agenesis [4]. Patients with ICA agenesis are at an increased risk of developing an aneurysm in cerebral vessels [5]. The prevalence of cerebral aneurysms in the general population is 1 to 5%; however, the risk for patients with ICA agenesis is estimated at 24 to 34% [1][2][3]. This increased risk is believed to be because of hemodynamic stress and abnormal flow through collateral circulations in these patients [6].

Aplasia, hypoplasia, and agenesis of the ICA are rare congenital anomalies, with less than 200 cases reported worldwide [7]. Some authors believe that the true incidence in the general population is higher as most cases are clinically asymptomatic [6]. The term agenesis is referred to the total absence of the entire artery due to an embryological arterial developmental failure; however, the terms hypoplasia and aplasia are applied to describe the situation when a portion or remnant of the artery is present and when the initial segment of the artery is normal in size or even slightly enlarged proximal to its abrupt narrowing [8].

As endovascular interventions become more widespread in thromboembolic events, recognizing anomalies in the brain circulating system is highly important. In some previous studies, unilateral ICA agenesis was diagnosed incidentally in magnetic resonance angiography (MRA) and/or computed tomography (CT) angiography. Hence, we present two rare cases of asymptomatic unilateral ICA agenesis that were suspected in carotid ultrasound study.

## Case Presentation

**Figure-1:**
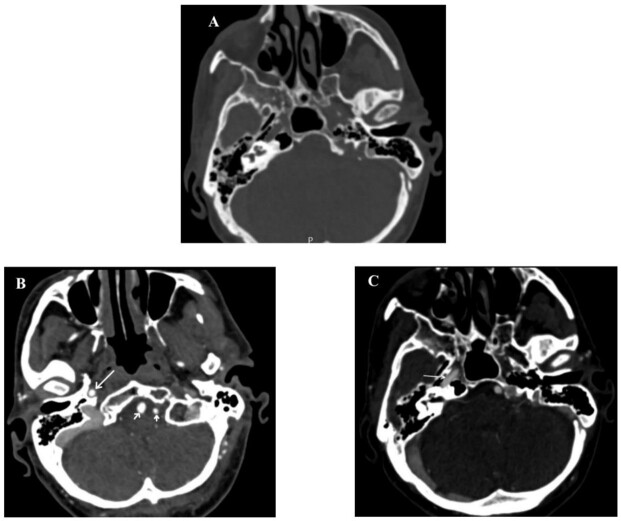
Figure 1. A: Non-enhanced axial CT image in case 1 shows the absence of the left petrous carotid canal within the skull base. B: Axial CT angiography image at the level of the skull base in case 1 shows the ICA (long arrow) and bilateral vertebral arteries (short arrows); however, the left ICA is absent. C: Just superiorly, the right ICA is present within its carotid canal (white arrow); however, the left ICA and left carotid canal are absent.

### Case 1

The patient was a 63-year-old Iranian man with the three-vessel disease who was referred to our cardiovascular hospital (Farshchian, Hamadan, Iran) for coronary artery bypass graft (CABG) surgery. He had no previous history of diseases except right arm numbness lasting about 15 minutes six months ago. Routine laboratory tests were within normal limits. His electrocardiogram (ECG) and chest X-ray were also normal. The patient was referred to our radiology department for ultrasonographic evaluation of extracranial arteries with suspicion of a previous transient ischemic attack (TIA). In color Doppler ultrasonography of carotid arteries, the right common carotid artery (CCA) bifurcation location was lower than normal. An echogenic plaque measuring 14.6×1.8 mm was noted at the proximal portion of the right ICA without hemodynamic changes. The CCA bifurcation, external carotid artery (ECA), and ICA were not visible on the left side. No remarkable finding was found in the left CCA except a small echogenic plaque. Flow patterns in both vertebral arteries were normal.

The patient underwent cervical and brain arteries multidetector CT angiography with intravenous administration of 90 ml of nonionic water-soluble contrast media (Visipaque, 320 mg/ml), using low dose 128-slice multidetector CT scanner (Siemens, SOMATOM Definition AS, Germany). After manual selection of the field of view, data was reconstructed, keeping slice thickness 5 mm, and reconstructed increment (0.6 mm) in dedicated soft tissue kernel setting, and it was analyzed in axial, sagittal, and coronal views. Multiplanar reformation, maximum intensity projection with bone removal, and volume rendering images were reconstructed. The left ICA and its bony carotid canal were absent (Figure-1). Both CCA and ECA showed patent lumen and good luminal flow without evidence of stenosis. Both vertebral arteries were well-developed. The A1 segment of the left anterior cerebral artery was hypoplastic. Other findings in the neck and/or brain were unremarkable. A CABG was performed, and the patient was discharged without any complications. He was well three months after surgery.

### Case 2

A 69-year-old Iranian woman with chest pain radiating to her back and a previous history of hypertension, diabetes, and cholecystectomy was referred to our cardiovascular hospital. The patient received antihypertensive medication, i.e., losartan 25 mg/dl every 12 hours, and antidiabetic medication (metformin, 500 mg/dl every 12 hours). Also, she reported a history of a slurred speech lasting about 30 minutes three months ago. The patient's blood pressure was measured at 140/90 mmHg. The routine laboratory tests revealed an increase in troponin and CK-MB; however, other parameters were within normal limits. She had undergone coronary angiography, which revealed the three-vessel disease. She was a candidate for CABG and was referred to our radiology department for ultrasonographic evaluation of extracranial arteries with suspicion of a previous TIA.

In an ultrasound examination, the length of the left CCA was 3.6 mm, which suggests a small CCA. Bifurcation of the left CCA was not seen, and the spectral waveform of the left CCA was highly resistant, resembling the right ECA. Other findings were unremarkable.

The patient underwent spiral CT angiography of the carotid and brain arteries for further evaluation (Figure-2). The C1 to C7 and A1 segments of the left ICA and its canal in the left temporal bone were absent, suggesting unilateral ICA agenesis. At the circle of Willis, the left middle cerebral artery (MCA) originated from the P1 segment of the right posterior cerebral artery (PCA). Moreover, the right P2 and P3 segments of PCA originated from the right ICA (fetal origin). Other findings were unremarkable. A CABG was performed, and the patient was discharged without any complications. She was well three months after surgery.

## Discussion

**Figure-2:**
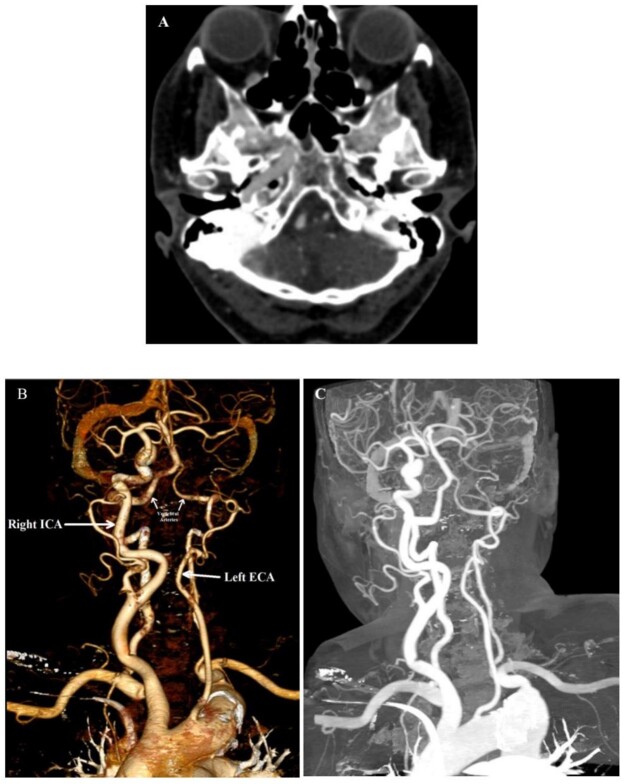
Figure 2. A: Axial CT angiography image at the level of skull base shows the absence of left ICA and left petrous carotid canal. B: Coronal volume rendered image and maximum intensity projection view of cranial vasculature (C) in case 2 revealed the absence of left ICA.

### Etiology

The cause of ICA agenesis is currently unknown, and no definite risk factors are detected for its occurrence. The malformation is present from birth and is believed to be caused when something happens early during the development of the baby that stops the carotid artery from forming correctly [5]. Exaggerated folding of the embryo toward one side and constriction by the amniotic band can lead to ICA agenesis [5]. Mostly,malformation is thought to occur by chance. However, less commonly, a person with ICA agenesis may also have other diseases [3]. Patients with ICA agenesis caused by other syndromes typically have other symptoms and/or medical problems [1][2][3]. Agenesis of ICA may be unilateral or bilateral. There are 27 cases of bilateral ICA agenesis in the literature, suggesting that this developmental disturbance usually occurs unilaterally [3].

### Inheritance and Epidemiology

ICA agenesis has a male predominance and has an increased incidence on the left side with a reported ratio of cases of 3:1 [7]. Consistently, both of the patients in our study had left side involvement. The true incidence of ICA agenesis is unknown because most cases are asymptomatic and are found incidentally [8]. In a retrospective review of cerebral magnetic resonance imaging (MRI) and MRA, Ryan et al. [9] found seven patients with either absence or hypoplasia of the ICA from more than 5000 examinations, with an incidence of 0.13%.

Mutations in a specific gene have not been associated with ICA agenesis, and the malformation is not known to run in families [4]. Therefore, other family members are not known to be at risk of having the malformation. However, for the individual with ICA agenesis who has an aneurysm, other family members may be recommended to have screening to check for aneurysms as >well [10]. ICA agenesis is associated with a few other diseases and/or syndromes [3]. Among individuals with ICA agenesis as the sign of another disease or syndrome, it is possible that the malformation is inherited and can be passed on to future generations. The long-term outlook for patients affected by ICA agenesis is typically good.

Zink et al. [11] found that 27.8% of cases involving carotid agenesis or hypoplasia are associated with intracerebral aneurysms, compared to an incidence of 2 to 4% in the general population. Indeed, the increased incidence of cerebral aneurysms for patients older than 30 years suggests that cerebral aneurysms are acquired rather than congenital [11]. Other clinical symptoms that have been reported in the setting of carotid artery agenesis include pulsatile tinnitus, TIA symptoms, migraine, and Horner’s syndrome [12].

Rare syndromes, such as posterior fossa brain malformations, hemangiomas, arterial lesions, cardiac abnormalities/aortic coarctation and eye abnormalities, Goldenhar syndrome, coarctation of the aorta, and Klippel-Feil syndrome have been reported in the setting of carotid agenesis [13][14][15][16]. Other endocrinologic deficiencies have also been reported in carotid ageneses, such as hypopituitarism and growth hormone deficiency [17][18][19].

### Diagnosis and Collateral Circulation

Diagnosis of ICA agenesis often occurs accidentally when a person performs a brain MRI and/or CT scan. Important findings necessary for ICA agenesis diagnosis include the absence of the carotid canal at the skull base, the absence of the ipsilateral ICA, and the hypoplasia of ipsilateral CCA [20]. Mentioned criteria and patients' clinical signs and symptoms differentiate ICA agenesis from occlusion by thrombus or emboli, moyamoya disease, and ICA vasospasm [20]. Ultrasonographic evaluation of the carotid arteries can arise suspicion about this

entity [21]. The diagnosis can be confirmed with MRA and/or CT angiography [22].

Demonstration of a normal bony carotid canal effectively rules out developmental ICA agenesis [22]. The bony carotid canals were absent in both cases presented here, suggesting ICA agenesis. Collateral circulation in patients with agenesis of ICA is typically from the contralateral ICA and the vertebrobasilar system via the circle of

Willis [23]. Collateral circulation in ICA agenesis seems to depend on when the injury to the ICA embryologic origin occurs. For instance, an insult to ICA origin after the development of the basilar artery but before the completion of the Willis circle would probably result in collateral circulation via this circle [7]. Six types of collateral circulation are established in the case of unilateral ICA agenesis by Lie et al. [8]:

(A): Collateral circulation to the ipsilateral anterior cerebral artery (ACA) through a patent anterior communicating artery (ACOM) and ipsilateral MCA through the hypertrophied posterior communicating artery (PCOM) in a patient with unilateral ICA agenesis.

(B): Collateral circulation to ipsilateral ACA and MCA through a patent ACOM in unilateral ICA agenesis.

(C): Bilateral ICA agenesis with supplies to the ACAs and MCAs through the PCOMSs.

(D): Unilateral agenesis of the cervical portions of the ICA with collateral from anintercavernous communication from the cavernous segment of the contralateral ICA.

(E): Hypoplasia of bilateral ICA with diminutive ACAs and prominent PCOMs. In this type, most blood flow in the MCAs originates from posterior circulation.

(F): Collateral flow to distal ICA via the internal maxillary branches of the ECA.

Evaluating collateral pathways in patients with unilateral ICA agenesis is clinically important because in cases with a lack of collateral flow from the circle of Willis, the patients would be susceptible to injury in the cerebral hemisphere ipsilateral to the absent ICA. For example, in the subtype D of the Lie et al. classification system, occlusion of ICA could potentially cause infarction in the bilateral MCA territories. In both cases of our study, collateral circulation to ipsilateral ACA and collateral circulation to ipsilateral MCA were through ACOM and PCOM, respectively, which is compatible with subtype A of the Lie et al. classification system. This collateral circulation pattern is the fetal variant and is the most common type of collateral circulation in patients with ICA agenesis [24]. A1 segment of ACA ipsilateral to the ICA agenesis was hypoplastic in both cases, consistent with previous findings in patients with this anomaly [25].

### Management

Given the asymptomatic and congenital nature of ICA agenesis, no treatment is necessary for these patients. Moreover, there is no difference in the management of carotid stenosis between patients with ipsilateral or contralateral ICA agenesis and the general population. ICA agenesis may comprise an additional trigger factor for the development of significant comorbidities [4]. Different previous studies stress the importance of conducting periodic imaging studies of the cerebral vessels of patients with ICA agenesis, with the objective of screening for the development of intracranial aneurysms [26]. Early recognition of anomalies in the carotid system can prevent potentially fatal complications; hence, MRA has been recommended to screen and monitor aneurysms [26]. However, because of the rarity of ICA agenesis and the paucity of studies in this field, experiences from these studies cannot be conclusive to design a comprehensive guideline.

## Conclusion

Herein, two cases of asymptomatic unilateral ICA agenesis were presented to our hospital due to coronary artery disease. Suspecting ICA agenesis at color Doppler imaging of the neck and differentiating it from occluded ICA at CT angiography is important for correct diagnosis and management of the patients.

## Conflict of Interest

The authors have nothing to disclose.
